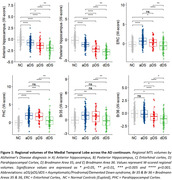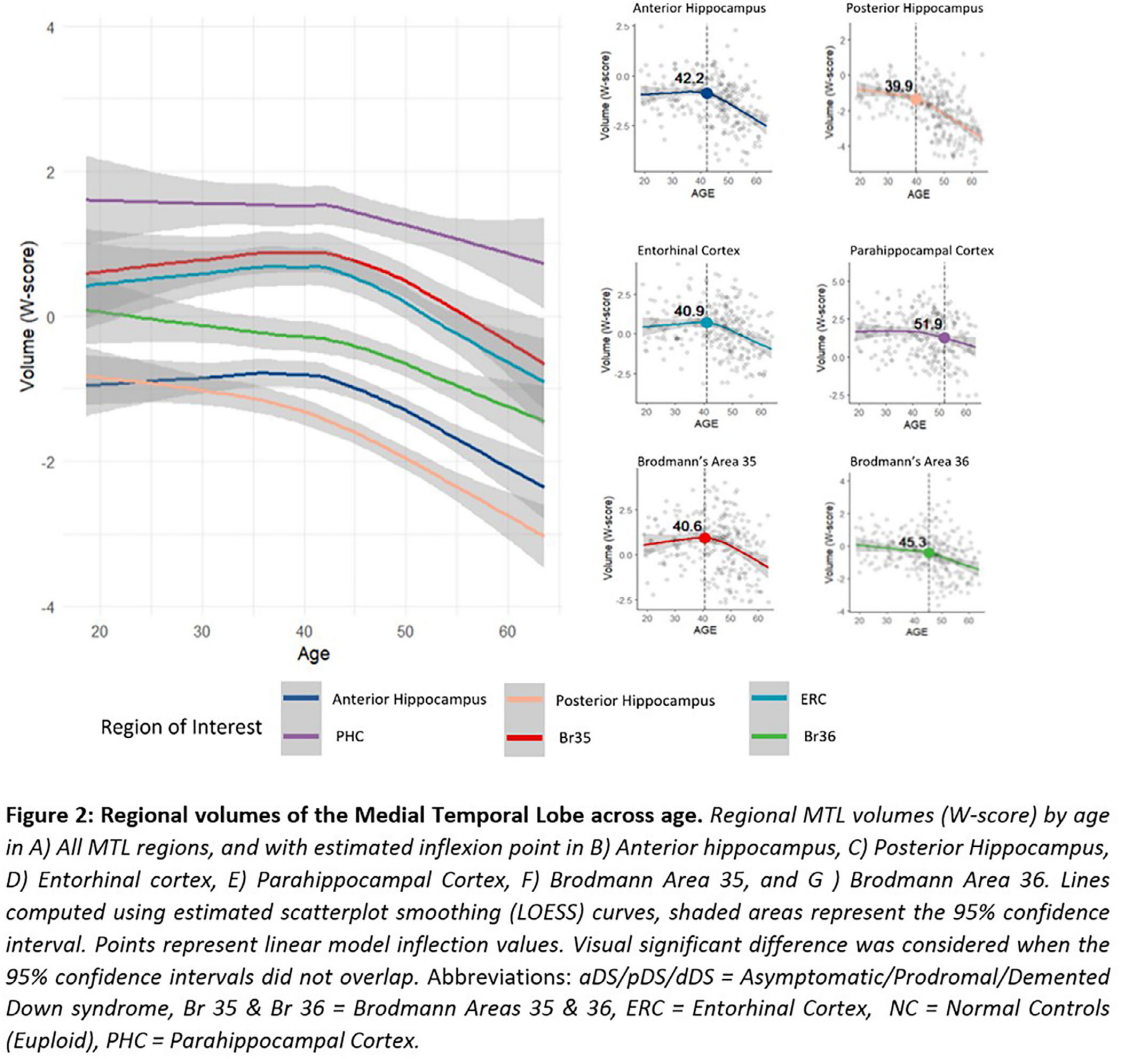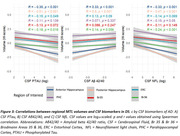# Volumetric Characterization of Medial Temporal Lobe in Down Syndrome Along the Alzheimer's Disease Continuum

**DOI:** 10.1002/alz.092133

**Published:** 2025-01-09

**Authors:** Sara E Zsadanyi, Bejamin J. Buehner, Alejandra O. Morcillo‐Nieto, Mateus Rozalem Aranha, José Enrique Arriola‐Infante, Lídia Vaqué‐Alcázar, Javier Arranz, Íñigo Rodríguez‐Baz, Lucía Maure‐Blesa, Laura Videla, Isabel Barroeta, Laura Del Hoyo, Bessy Benejam, Susana Fernandez, Aida Sanjuan Hernandez, Núria Bargalló, Sofía González‐Ortiz, Sandra Giménez, Robin de Flores, Daniel Alcolea, Olivia Belbin, Alberto Lleo, Maria Carmona‐Iragui, Juan Fortea, Alexandre Bejanin

**Affiliations:** ^1^ Sant Pau Memory Unit, Hospital de la Santa Creu i Sant Pau, Biomedical Research Institute Sant Pau, Universitat Autònoma de Barcelona, Barcelona Spain; ^2^ Sant Pau Memory Unit, Hospital de la Santa Creu i Sant Pau, Biomedical Research Institute Sant Pau, Universitat Autònoma de Barcelona, Barcelona, Barcelona Spain; ^3^ Department of Medicine, Faculty of Medicine and Health Sciences, Institute of Neurosciences, University of Barcelona, Barcelona, Spain. Institut d’Investigacions Biomèdiques August Pi i Sunyer (IDIBAPS), Barcelona Spain; ^4^ Hospital de la Santa Creu i Sant Pau ‐ Biomedical Research Institute Sant Pau ‐ Autonomous University of Barcelona, Barcelona, Catalonia Spain; ^5^ Center of Biomedical Investigation Network for Neurodegenerative Diseases (CIBERNED), Madrid Spain; ^6^ Barcelona Down Medical Center, Fundació Catalana Síndrome de Down, Barcelona Spain; ^7^ Radiology department, Centre de Diagnostic per la Imatge. Hospital Clínic de Barcelona, Barcelona Spain; ^8^ Multidisciplinary Sleep Unit, Hospital de la Santa Creu i Sant Pau, Barcelona Spain; ^9^ Normandie Univ, UNICAEN, INSERM, U1237, PhIND "Physiopathology and Imaging of Neurological Disorders", NeuroPresage Team, GIP Cyceron, Caen France; ^10^ Centre of Biomedical Investigation Network for Neurodegenerative Diseases (CIBERNED), Madrid Spain

## Abstract

**Background:**

Individuals with Down Syndrome (DS) almost invariably develop Alzheimer's Disease (AD), but detecting early clinical changes is challenging due to comorbid intellectual disability, highlighting the importance of non‐invasive biomarkers. Neuroimaging of the medial temporal lobe (MTL), a key site of tau pathology, shows promise as an early AD biomarker. Here, we aimed to characterise volumetric patterns of the MTL in DS across the AD clinical continuum, and define associations with AD cerebrospinal fluid (CSF) biomarkers.

**Method:**

253 adults with DS and 190 euploid controls from the Down Alzheimer Barcelona Neuroimaging Initiative underwent a 3T‐MRI protocol, and a comprehensive clinical assessment. T1‐weighted images were used to parse the medial temporal lobe using the Automated Segmentation of Hippocampal Subfields (ASHS) pipeline. Segmentation quality was visually inspected and W‐scores were computed for MTL subregions (anterior and posterior hippocampus, entorhinal cortex (ERC), parahippocampal cortex (PHC), and Brodmann areas Br35 and Br36) to adjust volumes for total intracranial volume, age and MRI scanner. Non‐parametric statistical tests were employed to assess volumes by AD clinical stage, age, and CSF biomarkers of AD.

**Result:**

Hippocampal and Br36 volumes gradually decreased with AD clinical stage, and all subregions were decreased at the dementia stage (dDS) compared to asymptomatic (aDS) and prodromal (pDS) stages (Figure 1). Surprisingly, significantly larger ERC, PHC and Br35 volumes were found at the asymptomatic DS stage compared to controls. All subregions had decreased volumes with age, with inflexion points around 40y for the hippocampus, ERC and Br36, 45y for Br35 and 50y for PHC (Figure 2). Most subregions exhibited significant correlations with CSF Aβ_42/40_ ratio, p‐tau‐181 and neurofilament light chain, and the strongest associations were found with anterior and posterior hippocampus (Figure 3).

**Conclusion:**

AD clinical stage and age are associated with progressive decreasing MTL volumes. Among all subregions, the hippocampus correlated best with CSF measures and appears particularly sensitive to detect early disease processes. These results indicate effectiveness of MTL volumes as a biomarker of early AD pathological changes in DS. Further studies are required to determine the pathological substrate of MTL atrophy and understand the increased volumes in some subregions.